# The Lipid Metabolic Landscape of Cancers and New Therapeutic Perspectives

**DOI:** 10.3389/fonc.2020.605154

**Published:** 2020-12-08

**Authors:** Wenjun Wang, Ling Bai, Wei Li, Jiuwei Cui

**Affiliations:** Cancer Center, The First Hospital of Jilin University, Changchun, China

**Keywords:** cancer, cancer metabolism, fatty acid catabolism, cholesterol, fatty acid synthesis, lipid uptake, tumor microenvironment

## Abstract

Lipid metabolism reprograming, as a hallmark of malignancy, has received renewed interest in recent years in such areas as energy sources, cell membrane components, and signaling molecules involved in the rapid tumor growth and the adaptation to the tumor microenvironment. Lipid metabolism deregulation in cancer involves multiple aspects, including an increased lipid uptake, endogenous *de novo* fatty acid synthesis, fatty acid oxidation, and cholesterol accumulation, thereby promoting tumor growth and progression. Recent advances in the understanding of specific metabolic alterations in cancer reveal novel pathogenesis mechanisms and a growing number of drugs targeting lipid metabolism have been applied in anti-tumor therapy. Thus, this review discusses the lipid metabolic landscape of cancers and the interplay with oncogenic signaling, and summarizes potential therapeutic targets to improve the therapeutic efficiency in cancer patients, in order to provide more reference and thinking for the treatment of lipid metabolism of cancer patients.

## Background

Lipid metabolism reprograming is a hallmark of cancer and plays an important role in shaping the tumor microenvironment and cancer cell phenotype, contributing to the occurrence and development of tumors (1). Lipid metabolism of tumor cells can be used to store energy and act as a mediator for cell signaling cascades by utilizing carbon-based precursors produced by aerobic glycolysis to synthesize basic cell components necessary for proliferation (2). Thus, lipid metabolism reprogramming is an essential link in tumor metabolism.

Lipids include triglycerides (TGs), phospholipids (PLs), sphingolipids, and cholesterol, and can function as energy sources, cell membrane components, and precursors of molecules involved in multiple biological processes (such as steroid hormones, vitamins, bile acids, and eicosanoids). Changes in lipid metabolism can affect numerous cellular processes, including cell proliferation, differentiation, and motility (3). In addition, since cancer cells compete for oxygen and nutrients in a nutrient-limited microenvironment, they maintain their malignant potential by altering their metabolism and obtaining fatty acids (FAs).

Cancer cells rewire lipid metabolism through several mechanisms, including increased *de novo* synthesis and exogenous uptake of FAs, upregulated fatty acid oxidation (FAO), cholesterol accumulation, and induced cancer-associated adipose tissue. At the same time, cancer cells metabolic reprograming can also influence immune cells in a variety of ways, such as through metabolic competition, oncometabolites, and exosomes (4, 5). With the gradual understanding of the extensive roles of metabolism in cancer pathogenesis, the specific metabolic preferences of cancer cells have been exploited to limit cancer progression for clinical benefit, and some therapies have been tested in clinical trials addressing their efficacy in multiple cancers. Meanwhile, accumulating evidence indicates that metabolism-targeting combination treatment or short-term starvation can improve the immune therapy or chemotherapy efficacy (6). Based on the studies on lipid metabolism in pan-cancer, the most extensive changes in lipid metabolism pathways are FA metabolism, cholesterol metabolism, arachidonic acid metabolism, and peroxisome proliferator-activated receptor (PPAR) signal transduction (1). Therefore, this review mainly anchors new advances in FA metabolism and cholesterol metabolism in tumor cells, aiming to provide potential targets for novel cancer therapeutic options.

## FA Metabolism Deregulation Supports Cancer Progression

**Figure 1:**
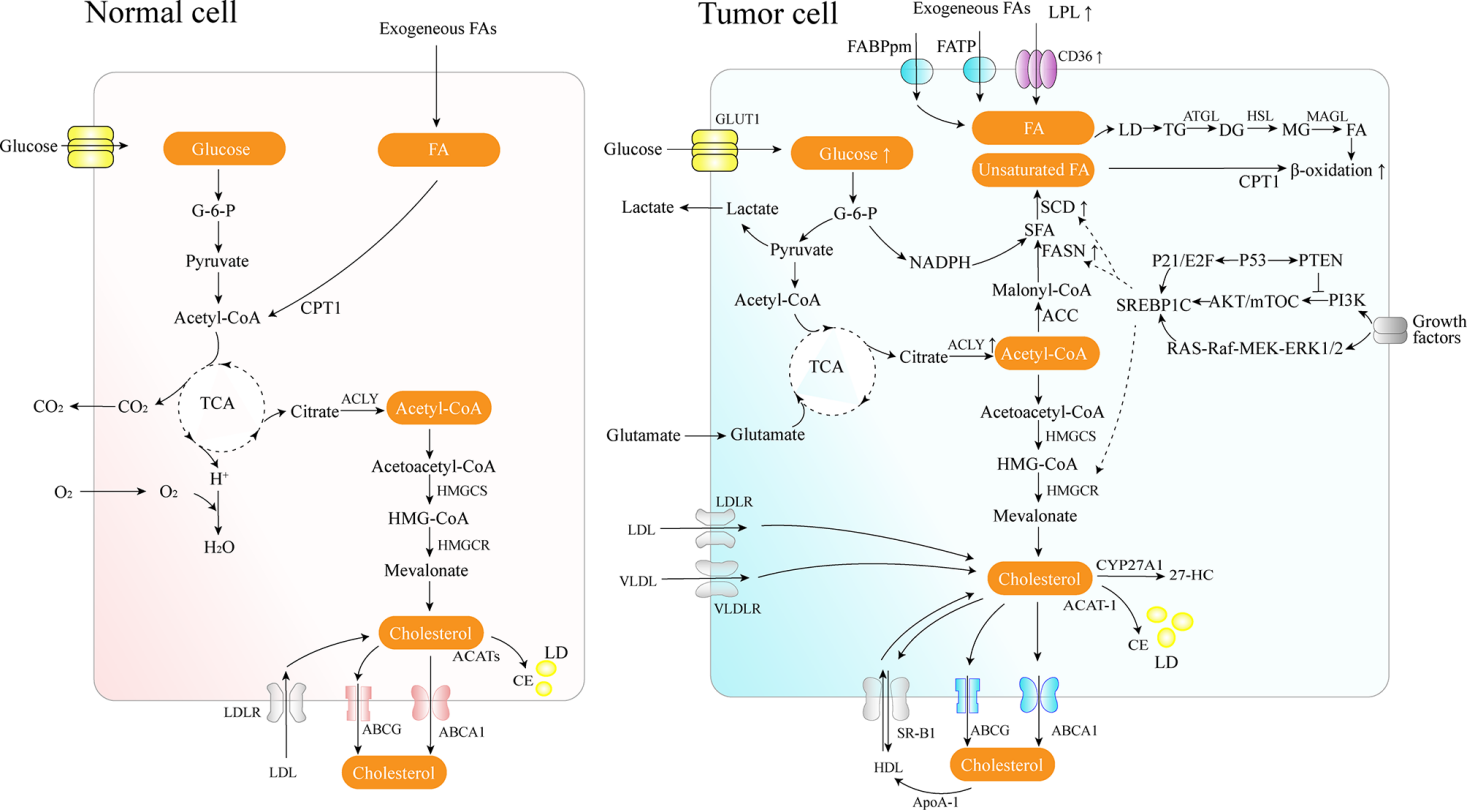
Lipid metabolism overview in normal and cancer cells. Cancer cells acquire diet-derived FA through LPL, CD36, FATPs, and FABPpm. Glucose is converted to acetyl-CoA by glycolysis and on to citrate through the TCA cycle in the mitochondria. The citrate is transported to the cytoplasm and converted back to acetyl-CoA by citrate lyase, which is used as the carbon source for the growing acyl chains. The pentose phosphate pathway from glycolysis generates NADPH. Cancer cells also develop effective *de novo* FAS machinery with an increase in the activity of key lipogenic enzymes. The surplus lipids (including excess FAs and cholesterol) in a cell exist in the form of neutral, inert biomolecules in the core of LDs. ATGL catalyzes the initial step of lipolysis, converting TGs to DGs; HSL is primarily responsible for the hydrolysis of DGs to MGs, and MAGL hydrolyzes MGs into FFA and glycerol. CPT1, as an outer mitochondrial membrane enzyme, translocates FA across the mitochondrial membranes and then the degradation of long-chain FAs occurs in the mitochondria. Cholesterol homeostasis involves the interplay between *de novo* synthesis (mevalonate pathway), uptake of dietary cholesterol, and removal of excess cholesterol from peripheral tissues. 27-HC is the metabolite substrate of cholesterol by CYP27A1 enzymes. SREBP-1 is activated through the PI3K/Akt/mTOR pathway and the Ras/Raf/MEK/ERK signaling pathway.

FAs are the crucial building blocks of several lipid species, and dysregulated FA metabolism is a vital component of lipid metabolism reprograming in cancer. Due to the critical roles of the FAs in the synthesis of the biological membrane, second messengers to transduce signals as well as vital energy sources (2). FA metabolism reprograming contributes to rapid proliferation and invasiveness of the tumor. Lipid metabolic reprograming in cancer cells includes several aspects, such as increased lipid uptake, *de novo* fatty acid synthesis (FAS), and FAO (**Figure 1**).

### Increased Lipid Uptake Benefits From Circulating FAs

Tumor cells can use circulating free FAs (FFAs) as an energy supply by lipolysis for membrane biosynthesis or signaling processes. Lipoprotein lipase (LPL), CD36 (also known as a fatty acid translocase, FAT), fatty acid transport protein family (FATPs), and plasma membrane fatty acid-binding proteins (FABPpm) are used to acquire diet-derived FAs from the bloodstream by lipolysis in specific cancer cells, such as non-small cell lung cancer (NSCLC), triple-negative breast cancer, liposarcoma, prostate cancer (PCa), etc. (7, 8). LPL is a crucial enzyme secreted by extracellular lipolysis and bound to the luminal surface of capillary endothelial cells, and it can potentially be supplied by tumor cells or by nonmalignant cells in the tumor microenvironment (8, 9). The enzyme is responsible for the TGs hydrolysis from circulating chylomicrons and very-low-density lipoprotein (VLDL). LPL is frequently overexpressed in invasive cervical squamous cell carcinomas, and subsequently increases its invasiveness (10).

FAs released by circulating TGs hydrolysis can be taken up by cells *via* CD36 for exogenous FFAs uptake, and CD36 exhibits a high affinity for transporting long-chain FAs (8, 11). Moreover, in recent decades, numerous studies have concluded that CD36 plays a role in accelerating tumor growth, metastasis, regulating chemoresistance and radioresistance, modulating tumor immunity, etc. (11–13). Oleic acid, the principal lipid in olive oil, can upregulate the expression of CD36 and facilitate tumor development by activating the Src kinase and the downstream ERK1/2 pathway in a CD36-dependent manner (14). In gastric cancer, CD36 promotes the uptake of exogenous palmitic acid to induce metastasis *via* the AKT/GSK-3β/β-catenin signaling pathway; therefore, targeting CD36 might constitute a promising new therapeutical approach for peritoneal metastases (12). Elevated CD36 levels and the consequent elevated FFAs uptake may activate the Wnt and TGF-β signaling pathways, thereby inducing epithelial-mesenchymal transition (EMT), which is involved in cancer cell metastasis (15). In pancreatic ductal adenocarcinoma, CD36 can also enhance the expression of several anti-apoptotic proteins, contributing to the resistance to gemcitabine and poor prognosis (13).

On other hand, tumor hypoxia constrains oxygen-dependent stearoyl-CoA desaturase (SCD), resulting in an accumulation of saturated FA precursors (16). While, in hypoxic condition, increased uptake of exogenous unsaturated lipids also plays a vital role in bypassing the requirement for FA desaturation, thus alleviating saturated FA-induced toxicity and maintaining homeostasis in hypoxic cancer cells (16).

### Upregulated Endogenous *de Novo* FAS Formation Accommodates the Increased Demand for Lipids

*De novo* FAS in the adult organism occurs mainly in the adipose tissue, liver, and the lactating breast, and people consuming an adequately balanced diet present little endogenous FAS (17). However, several cancer cells show high rates of *de novo* endogenous FAS (18). In addition, when tumors grow in reduced blood vessel density areas, access to lipids in the circulatory system is also reduced.

FAs are essential constituents of all biological membrane lipids and are crucial substrates for energy metabolism. Since tumor cell growth and division demand carbon, nitrogen, free energy, and reducing equivalents from glucose and glutamine, aerobic glycolysis can support the robust production of acetyl-CoA and nicotinamide adenine dinucleotide phosphate (NADPH) needed for FAS (19). Long-chain saturated FAs can be further modified by elongases or desaturases to form more complex FAs, which are used to synthesize various cellular lipids such as PLs, triglycerides, and cholesterol esters, or to acylate proteins (18). Cancer cells develop capable *de novo* FAS machinery with an increase in the activity of key lipogenic enzymes such as adenosine triphosphate (ATP)-citrate lyase (ACLY), acetyl-CoA carboxylase (ACC), CoA carboxylase (ACACA), fatty acid synthase (FASN), and SCD (18).

In the cytosol, ACLY converts mitochondria-derived citrate into acetyl-CoA, a vital building block for the endogenous biosynthesis of FAs and cholesterol. ACLY presents elevated levels of activity and expression in several types of cancers (20). In contrast, ACLY knockdown may trigger the activation of p53, thereby facilitating DNA damage-induced apoptosis in cancer cells (20). Moreover, the FAS pathway has two rate-limiting enzymes, ACC and FASN. ACC catalyzes the ATP-dependent carboxylation of acetyl-CoA, generating malonyl-CoA used for FA synthesis following the conversion of citrate and acetate to acetyl-CoA. FASN, downregulated in most normal human tissues, is the leading synthetic enzyme that catalyzes the NADPH-dependent condensation of malonyl-CoA and acetyl-CoA to produce the saturated FA palmitic acid. In contrast, FASN is often highly expressed in human cancers and represents a nearly universal phenotypic alteration in most human malignancies, such as breast, prostate, colon, ovary, endometrium, thyroid, esophagus, stomach, lung cancer, etc. (18, 21, 22). Additionally, a metabolic feature of PCa progression consist of increased rates of *de novo* FAS *via* overexpression of FASN, especially in metastatic castration-resistant prostate cancer (mCRPC) (23).

As master regulators of cholesterogenesis and lipogenesis, sterol regulatory element-binding proteins (SREBPs, including SREBP1a, SREBP1c, and SREBP2) transcriptionally activate a cascade of enzymes required for endogenous cholesterol, FAs, TGs, and phospholipid synthesis (24) (**Table 1**). In hepatocellular carcinoma (HCC), SREBP-1 promotes cancer cell proliferation and metastasis, and its levels negatively correlate with the HCC patient prognosis (25). Previous work has shown that SREBP-1 is activated through several mechanisms, including the phosphatidylinositol 3-kinase (PI3K)/Akt/mTOR pathway, Ras/Raf/MEK/extracellular signal-regulated kinase (ERK) signaling pathway (Ras/ERK pathway), and oncogenic BRAF signaling (26, 27). First, a lack of expression or mutation of the tumor suppressor gene, PTEN, has been clearly established in various types of tumors, and NADH accumulation in respiration-deficient cells leads to inactivation of PTEN and subsequent activation of the Akt survival pathway (28). It has previously been reported that p53-mediated transactivation can also increase PTEN levels, as it has a functional p53 binding site within its promoter (29). Akt-dependent lipogenesis requires a mammalian target of rapamycin (mTORC1) activity to regulate SREBP1 activity, inducing the increased expression of enzymes involved in lipid biosynthesis, including ACLY, FASN, and ACC (30). Furthermore, in glioblastoma cells, epidermal growth factor receptor (EGFR) can promote SREBP-1 cleavage and nuclear translocation through EGFR-PI3K-Akt signaling, without depending on mTORC1 activity (31). In addition, hypoxia also has an impact on the level of FASN expression *in vivo*: hypoxia significantly upregulates SREBP-1 through induction of Akt, which then firmly binds to the SREBP-binding site/E-box sequence on the FASN promoter (32). Second, in BRAF^V600E^-mutant melanoma, SREBP-1 is a crucial downstream target of BRAF signaling that induces lipogenesis and enhances membrane lipid saturation, promoting targeted therapy resistance (26). In addition, SREBP-1 is also known to regulate glucose and glutamine metabolic pathways, and SREBP-1 can also protect tumor cells by enhancing glycolytic activities (25).

**Table 1:** Summary of transcription factors that regulate key enzymes in lipid metabolism.

Targets	Transcription factors	References
CD36	Peroxisome proliferator-activated receptors (PPARs) CCAAT/enhancer-binding protein (C/EBP), signal transducer and activator of transcription 3 (STAT3), liver X receptor (LXR), pregnane X receptor (PXR), forkhead box O1 (FoxO1), and hypoxia inducible factor 1 (HIF-1α)	(11)
ACLY	Sterol regulatory element-binding protein 1c (SREBP1c) carbohydrate response element-binding protein (ChREBP),	(96)
FASN	SREBP1c, ChREBP,	(96)
ACC	SREBP1c, ChREBP	(96)
SCD1	SREBP1c, ChREBP	(96)
HMGCR and HMGCR	SREBP-2	(97)
ABCA1	LXR and retinoid X receptor (RXR)	(98)

An ever-growing body of experimental evidence supports the notion that the oncogenic nature of FASN-associated lipogenesis strictly depends on the activity and/or expression of important oncogenes and tumor suppressors, such as *p53, MYC, HER2*, and retinoblastoma (*RB*). *MYC*, a dominant oncogene, in collaboration with SREBP, induces lipogenesis both *in vitro* and *in vivo*, and plays a role in the initiation and maintenance of tumorigenic growth in *MYC*-driven cancers (33). Rueda–Rincon et al. reported that p53, an important tumor suppressor, can repress SREBP1 expression *via* the p21 (cyclin-dependent kinase inhibitor 1A)/Rb/E2F transcription factor pathway, resulting in SCD and a subsequent decrease in mono-unsaturation of phospholipid acyl chains (27). *HER2*, which is frequently overexpressed in breast cancer and other cancers, can stimulate the expression of FASN *via* the PI3K/Akt/mTOR and Ras/Raf/MAPK pathway (34, 35). The prolyl isomerase Pin1 can enhance FAS by regulating ACC1, FASN, and the SREBP-1, and it can also suppress AMPK phosphorylation to stabilize the ACC1 protein (36).

Humans lack the enzymes required for generating polyunsaturated FAs from saturated and mono-unsaturated species; however, SCD1, which introduces a double bond in the Δ9 position of saturated fatty acids (SFAs) to produce mono-unsaturated fatty acids (MUFAs), has an increased expression in various cancer cells and is involved in the promotion of cancer cell proliferation, migration, metastasis, and tumor growth (37, 38). Intensively proliferating cancer cells are distinguished by the higher demand for MUFAs, which are utilized mainly to synthesize new membrane-forming PLs, triacylglycerols, and cholesteryl esters (37). The unsaturated FAs promote the activation of NF-κB (nuclear factor κ-light-chain-enhancer of activated B cells), a well-known pro-tumorigenic driver, which in turn regulates the expression levels of SCD1 at transcriptional level (39). Single-cell imaging studies and mass spectrometry analysis showed that ovarian cancer stem-like cells (CSCs) (ALDH^+^/CD133^+^) had an higher degree of lipid unsaturation than non-CSCs (ALDH^−^/CD133^−^), mediated by lipid desaturases (including SCD1 and Δ6) (39). In turn, lipid desaturases play a role in the maintenance of cancer cell stemness (39). Furthermore, Lai et al. proposed a Wnt-SCD- lipoprotein receptor-related protein (LRP) loop in CSC-related tumor development (40). In rodent hepatic stellate cells and tumor-initiating stem cell-like cells, Wnt/β-catenin interacts with SREBP-1c in novel sterol-regulatory element sites to promote SCD1 expression, which amplifies the Wnt pathway *via* stabilization of low-density LRP5 and six mRNAs (40).

### Increased Mitochondrial FAO Pathway

Long-chain FAs degradation occurs in mitochondria and is catalyzed by several carnitine acyl transferases, including carnitine palmitoyltransferase (CPT) 1, located in the outer membrane, and CPT2, located in the inner membrane, together with a carnitine-acylcarnitine translocase (CAT) (41). CPT1, as an outer mitochondrial membrane enzyme, catalyzes the rate-limiting step of β-oxidation by translocating FA across the mitochondrial membranes. Its activities vary according to tissue-specific needs in FA metabolism and energy expenditure. Immunohistochemistry staining has shown elevated expression levels of CPT1 in human glioma tissues (41).

Furthermore, tumor cells can acquire FAs through lipolysis to perform FA β-oxidation (also known as FAO), which further promotes cancer proliferation, survival, drug resistance, and stemness (42–44). FAO can be utilized to produce high levels of ATP and support the proliferation of triple-negative breast cancer, and glioma (41, 43). However, FAO is not a predominant oxidative substrate for ATP generation. Guppy and colleagues reported that approximately 10% glucose, 14% glutamine, 7% palmitate, 4% oleate, and 65% from unidentified sources contribute to the oxidative component in MCF-7 breast cancer cell line (45). Moreover, promyelocytic leukemia can modulate PPAR signaling and FAO, thereby contributing to the hematopoietic stem cell maintenance (44). At the same time, NANOG, a key regulator of cell reprograming, reduces mitochondrial oxidative phosphorylation (OXPHOS) and production of ROS and promotes FAO, contributing to the self-renewal ability and therapeutic of CSC (46).

### Flexible Regulation of Lipolysis and Lipophagy to Liberate Stored FAs

Considering that newly synthesized FAs are rapidly incorporated into neutral and phospholipid stores, cancer cells are also required to possess a complementary lipolytic pathway to liberate stored FAs for metabolic and signaling purposes (47). During periods of hypoxia, FA delivery to the cells exceeds FA oxidation rates, which consumes significant amounts of oxygen, resulting in elevated mitochondrial ROS production and subsequent cell damage and apoptosis, known as lipotoxicity (48, 49). Therefore, FA oxidation switch-off combined with the storage of excess FAs in TG-LDs through inhibition of lipolysis would constitute a conceivable strategy for cancer cells in hypoxia. The surplus lipids (including excess FAs and cholesterol) in a cell exist in the form of neutral, inert biomolecules in the core of lipid droplets (LDs) (50), which are a hallmark of hypoxic cancer cells, and are released through a combination of lipolysis and a selective autophagic mechanism called lipophagy (48, 51–53). At the same time, when exposed in acid TME, autocrine TGF-β2 signaling is drived in cancer cells, thus further fueling FAs uptake and oxidation as well as formation of LDs (54). LDs accumulation is associated with a more aggressive cancer phenotype and increased migration *via* elevated levels of pro-oncogenic signaling lipids, particularly lysophosphatidylcholines, and activation of pro-oncogenic SRC kinase signaling (53). Moreover, prolonged nutrient deficiency or lipid overload tend to provoke the autophagy of cancer cells (55). Cancer cells can employ LDs to modulate autophagy through providing the lipid precursors for the formation of autophagic membranes or signaling that activates the autophagy genes expression (56–58). *HER2* overexpression can upregulate the PPAR-γ, which promotes conversion and storage of excess FAs to TGs, thus allowing cells to avert cell death resulting from endogenous palmitate-related lipotoxicity (34). TGs can also sequester exogenous unsaturated FAs, particularly oleate (16). While, under serum- and O_2_-limited conditions, TGs can neutralize excess FA saturation through preferential release of unsaturated FAs to ameliorate stress (16).

Conversely, adipose triglyceride lipase (ATGL), hormone-sensitive lipase (HSL), and monoacylglycerol lipase (MAGL) provide a stream of intracellular FFAs that play important and critical roles in cancer cell proliferation and tumor progression by de-esterification.

ATGL has been found to be significantly reduced in a variety of human malignancies, including lung and pancreatic cancer. The role of ATGL in several cancer cells such as PCa and HCC is ambiguous, as contradicting evidence has been reported so far (59, 60). Liu et al. reported that ATGL is highly expressed in human HCC tissues and positively correlated with tumor size, predicting poor prognosis (61). At the same time, Di Leo et al. demonstrated that ATGL levels are inversely correlated with the proliferation rate of HCC-derived cell lines, which depend on intact ATGL enzymatic activity (62). Moreover, ATGL upregulation in breast cancer was associated with an enriched adipocyte tumor microenvironment (TME), contributing to the aggressiveness of high-grade tumors (63). Zhang et al. demonstrated that, in hypoxia, hypoxia-inducible gene 2 (HIG2), as a novel endogenous inhibitor of ATGL, mediates the lipolytic inhibition, promotes LD accumulation, attenuates ROS production, and enhances cancer cell survival (48). However, Di Leo et al. did not observe this mechanism in their model, suggesting that alternative mechanisms contributing to the control of proliferation need further research (62). In addition, long non-coding RNA NEAT1 expression is upregulated by hypoxia through hypoxia inducible factor (HIF)-2α in various types of cancers, and can disrupt hepatoma cells lipolysis *via* ATGL (61). Additionally, ATGL-mediated p53 acetylation by the PPARα/p300 axis is responsible for its inhibitory effect on glycolysis. In contrast, ATGL overexpression redirects HCC cell metabolism towards a less glycolytic phenotype *via* P53 and thereby is more resistant to glycolysis inhibitors (62). Liu et al. reported that ATGL contributed to the proliferation of HCC cells by upregulating AKT phosphorylation levels (64). Therefore, cancer cell metabolism is also regulated by direct crosstalk with tumor-surrounding stromal components (such as adipocytes) and hypoxia (63). On the other hand, ATGL may have a broad influence on cancer processes, such as redox homeostasis, inflammation, and autophagy, through PPARα signaling (59).

MAGL expression is highly elevated in human cancer cells and primary tumors, including PCa, neuroblastoma, HCC, colorectal, ovarian, endometrial cancers, etc. Aggressive cancer cells do indeed acquire the ability to liberate FFAs from neutral lipid stores because of the heightened expression of MAGL, which is the principal regulator of FFA levels (47). Zhang et al. confirmed that promoter methylation of large tumor suppressor kinase 1 (LATS1) resulted in the dysfunction of the Hippo signal pathway, which induced overexpression of MAGL in HCC (65). Moreover, Nomura et al. reported that MAGL regulates a host of secondary lipid metabolites that include essential signaling molecules, such as LPA and prostaglandin E2 (PGE2), which have been reported to promote cancer cell aggressiveness (47).

### Sphingolipid Metabolism Involves in Regulating Tumor Proliferation

Accumulating evidence indicates that sphingolipids including sphingosine, ceramide, and sphingosine-1-phosphate, involve multi-layered aspects of cancer cell biology (66). Perturbation of sphingolipid homeostasis has been reported in various solid tumors and hematological malignancies (67). As the most abundant cellular sphingolipids, ceramides can exert pro-apoptotic effects as tumor-suppressing lipids and augment the efficacy of chemotherapeutics and targeted therapies (67–69). However, several enzymes (e.g., acid ceramidase, ceramide kinase, etc.) that accelerate the metabolism of ceramides, have been found to have an enhanced expression level in multiple tumors (67, 68, 70). Therefore, ceramide metabolism has been utilized to develop some drugs that target these vital enzymes thus inducing lethal ceramide accumulation and overcoming cancer therapy resistance (67).

## Deregulation of Cholesterol Metabolism in Cancer

Cholesterol, as an essential component of the cell membrane, alters the biophysical properties of the membrane by governing its fluidity and impacts various biochemical functions, including modulation of signaling pathways and intercellular communication, as an integral component of lipid rafts (71, 72). Cholesterol homeostasis involves the interplay between *de novo* synthesis, uptake of dietary cholesterol (low density lipoprotein – LDL or high density lipoprotein – HDL), and removal of excess cholesterol from peripheral tissues (73) (**Figure 1**). In cancer, the reprogramed cholesterol metabolism can provide a signal transduction platform and activate stemness and oncogenic signaling (such as the Hedgehog pathway, mTORC1) as a second messenger or component of lipid rafts, thereby contributing to cancer progression and invasion (74).

### Regulation of Cholesterol Transport to Maintain Cholesterol Homeostasis

Since several tumor cells have been shown to present with an abnormal distribution of cellular cholesterol, such as clear cell renal cell carcinoma (ccRCC) and PCa bone metastases, some essential cholesterol transporters have been characterized as cancer-related factors (75, 76). As a transmembrane protein, the ATP-binding cassette transporter (ABCA1) is responsible for reverse cholesterol transport from the inner cell to the circulatory system. By stabilizing ABCA1, apolipoprotein A-I (ApoA-I) can recover the extracted cholesterol and synthesize HDL (77). The interaction of lipidated ApoA-I in discoid or more mature HDL particles with another transporter of the ABC family, ATP-binding cassette subfamily G member 1 (ABCG1), further contributes to reverse cholesterol transport. Finally, HDL particles binding to the scavenger receptor class B type 1 (SR-B1) transfer cholesterol down a cholesterol gradient (78). ABCA1 is significantly overexpressed, promotes EMT, and leads to increased invasiveness in advanced stages of colorectal cancer (CRC) (77). Reduced *ApoA-1* mRNA and protein levels have been found in HCC compared to normal liver tissue, the main source of ApoA-I (78). ApoA-I exhibits tumor suppressive activity both *in vitro* and in animal studies, accompanied by anti-inflammatory and immune-modulating effects, which have been reviewed by Georgila et al. (78). However, conflicting conclusions are present in some cancers; for example, ApoA-I levels were reported to be positively associated with breast cancer risk (79). As a receptor for the uptake of HDL-associated cholesterol, elevated SR-B1 expression causes an increased uptake of HDL-cholesterol in ccRCC through the reduction in CpG islands methylation (75). Besides, sterol carrier protein 2, an intracellular cholesterol trafficking protein, can transport cholesterol from the cytoplasm to the plasma membrane (80). Then, the membrane cholesterol concentration induces the activation of PKA/SUFU/GLI1 signaling *via* the smoothened receptor, which is well-known as Hedgehog signaling, resulting in the inhibition of apoptosis and promotion of the cell cycle in pituitary adenomas (80).

### Upregulated Cholesterol Synthesis

Cholesterol is synthesized *via* the mevalonate pathway and regulated by its rate-limiting enzyme, 3-hydroxy-3-methylglutarylcoenzyme A reductase (HMGCR), which catalyzes the reduction of 3-hydroxy-3-methylglutarylcoenzyme A (HMG-CoA) to mevalonate. In glioblastoma patients, CSCs have been found to overexpress mevalonate pathway genes, and MYC can alter mevalonate metabolism (81). A meta-analysis encompassing 865 primary breast cancer patients reported that high HMGCR and additional mevalonate pathway genes mRNA levels correlated with poor patient prognosis and reduced survival (82). Mevalonate was found to constitute an escape mechanism of survival and growth in HER2^+^ breast cancer models resistant to anti-HER2 therapies, partly through the activation of downstream YAP (Yes-associated protein)/TAZ (transcriptional coactivator with PDZ-binding motif)-Survivin signaling particularly through farnesyl pyrophosphate/geranylgeranyl pyrophosphate (83). Regulation of HMGCR is influenced by a network of modulatory proteins, including SREBP2, SREBP cleavage activated protein (SCAP), and insulin-induced gene (Insigs). Oncogenic growth signaling, such as PI3K/AKT and RAS/MAPK, is triggered to maintain cholesterol homeostasis by activating SREBP-mediated cholesterol biosynthesis. SREBP2, transcribed from the *SREBF2* gene, is the main transcription factor that activates the genes involved in mevalonate and cholesterol synthesis (84). Constitutively activated RAS/MAPK signaling also activates heat shock factor 1 (HSF1), thereby increasing the expression of cholesterol biosynthesis-related genes such as *SREBF2, HMGCR, HMGCS1*, etc. In contrast, HSF1 inhibition sensitizes HCC cells to the antiproliferative effects of simvastatin (85). Hypoxia also induces HIF-1α accumulation, which increases HMGCR levels and activity by stimulating its transcription (86). P53 can actively repress activation of SREBP-2 through transcriptionally upregulating the *ABCA1* (87). In breast cancer cells, mutant p53, which is the most frequent target for mutation in tumors, contributes to the upregulation of the mevalonate pathway, through recruitment of many sterol biosynthesis genes, or probably through one or more of the SREBP proteins (88). Furthermore, recent research reported that radiation upregulated the expression of four enzymes in the cholesterol biosynthesis pathway and increased the cell cholesterol content (89).

Moreover, cholesterol is also related to therapeutic resistance. Cholesterol and sphingolipids are also components of caveolae as major structural lipids, accompanied by the defining protein components, caveolins, and cholesterol appears to modulate caveolin-1 expression through a steroid regulatory binding element present in the caveolin-1 promoter and SREBP-1 (90). Since caveolin-1 is crucial for cellular energy homeostasis in tyrosine kinase inhibitor (TKI)-resistant tumor cells by mediating glucose uptake *via* GLUT3, Azhar et al. demonstrated a link between elevated cellular cholesterol and TKI resistance in NSCLC, which is independent of EGFR mutation status (91).

### Increased Cholesterol Metabolism and Esterification

The oxysterol 27-hydroxycholesterol (27-HC) is known as the metabolite substrate of cholesterol by cytochrome P450 family 27 subfamily A member 1 (CYP27A1) enzymes. An elevated level of serum cholesterol corresponds to a high level of serum 27-HC. As a selective endogenous estrogen receptor (ER) agonist and liver-X-receptor (LXR) agonist, 27-HC may contribute to ER-positive breast cancer growth and metastasis by inducing several EMT genes and disrupting constitutive p53 signaling in an MDM2-dependent manner (92, 93).

Furthermore, inside cells, the free cholesterol excess is esterified and stored as CE in LDs to prevent the toxicity generated by the over-accumulation of free cholesterol, mediated by acyl-CoA cholesterol acyltransferase (ACAT) encoded by *Acat1* and *Acat2* genes. Increased CE levels have been reported in breast cancer, leukemia, glioma, prostate, pancreatic cancer, renal cell carcinoma, etc. (94, 95).

## Therapeutic Targets for Cancer Treatment

**Figure 2:**
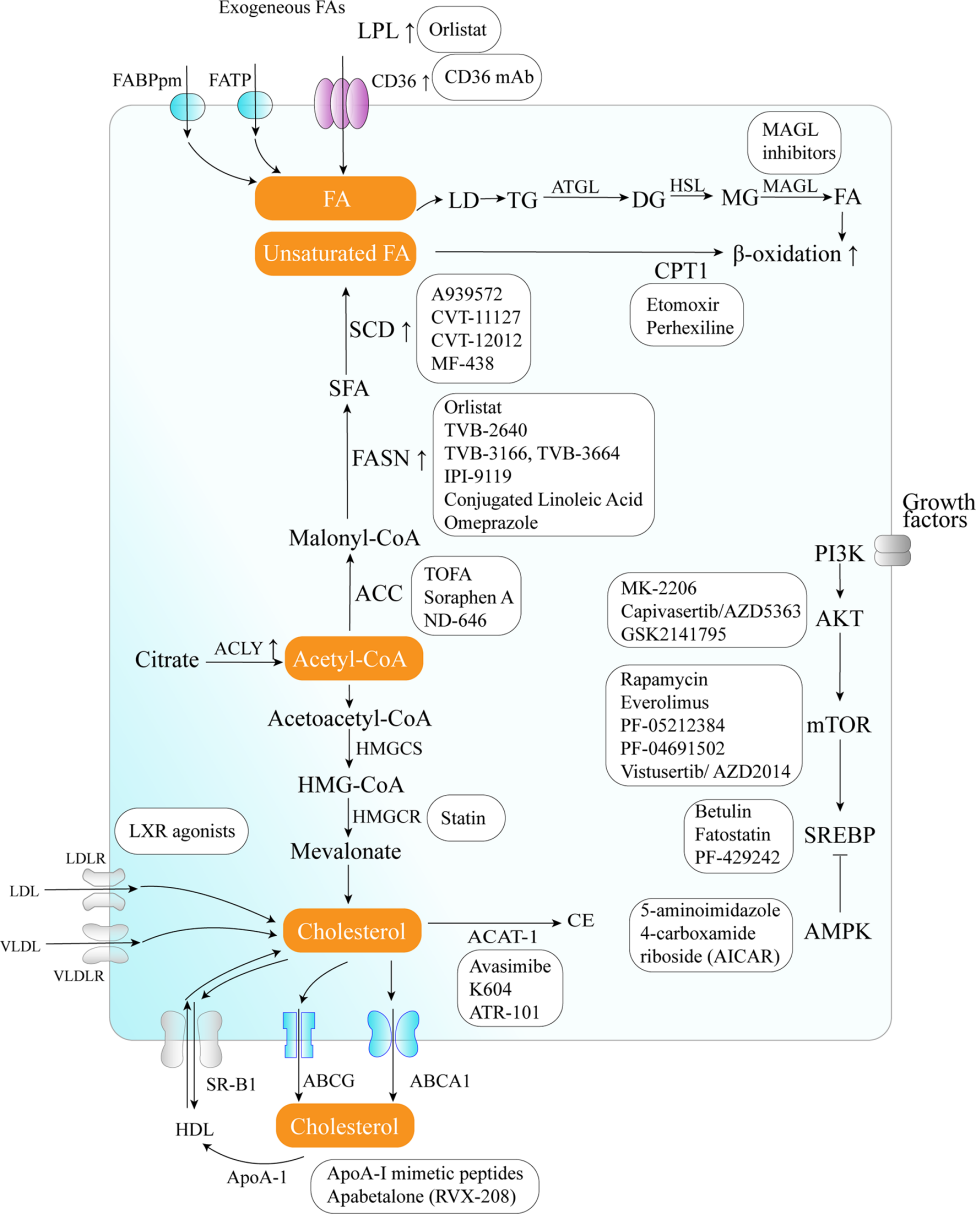
Therapeutic targets and anticancer drugs within the lipid metabolism pathway. The lipid metabolism-targeting therapies are shown as white boxes.

The critical role of altered metabolic pathways in cancer cell proliferation, but not in most normal human tissues, has led to some proposed strategies for cancer-specific treatments by targeting these pathways. To date, several metabolic enzymes inhibitors, such as glycolysis inhibitors, have been studied in clinical trials as targeted cancer therapeutics (**Table 2** and **Figure 2**).

**Table 2:** Therapeutic perspectives in lipid metabolic pathways.

Targets	Drug name	Type of cancer	Beneficial effects	Stage of development	References/clinical trial number
CD36	CD36 monoclonal antibody	Prostate cancer	Reduced fatty acid uptake and the abundance of oncogenic signaling lipids	Preclinical	(99)
FASN	IPI-9119	Castration-resistant prostate cancer	Selectively inhibit FASN and suppress expression of both full-length of androgen receptor (AR) and AR variant V7	Preclinical	(23)
	Orlistat	Prostate, breast, ovarian, colon cancer, and other solid tumors	An anti-obesity drug approved by FDA and an irreversible inhibitor of FASN	Preclinical	(100, 101)
	TVB-2640	Solid Malignant Tumor	A potent and reversible FASN inhibitor	Phase 2 Phase 2 Phase 1 Phase 1	NCT03032484 NCT03179904 NCT02980029 NCT02223247
	TVB-3166; TVB-3664	Oral squamous cell carcinoma, colorectal, breast cancer	A reversible and selective FASN inhibitor	Preclinical	(102, 103)
	Conjugated Linoleic Acid	Breast Cancer	Reduce *FASN* gene expression and spot 14	Early Phase 1	NCT00908791
	Omeprazole	Triple negative breast cancer	A proton pump inhibitors that can inhibit FASN	Phase 2	NCT02595372
ACC	TOFA	Lung cancer and colon carcinoma	Induce apoptosis as an allosteric inhibitor of ACC-alpha	Preclinical	(104)
	Soraphen A	Prostate cancer	Inhibit fatty acid synthesis and stimulate fatty acid oxidation	Preclinical	(105)
	ND-646	NSCLC	Inhibit fatty acid synthesis and tumor growth as an allosteric inhibitor of the ACC	Preclinical	(106)
mTOR	Rapamycin (<x>RAD001</x>)	Breast cancer	Inhibit S6 phosphorylation and cell proliferation, and resulted in lower levels of apoptosis induction	Preclinical	(107, 108)
	Everolimus	Castrated Resistant Prostate Cancer; Locally Advanced Cervical Cancer	Directly inhibit mTORC1 and indirectly inhibit mTORC2	Phase 3 Phase 1	NCT03580239 NCT01217177
	PF-05212384	Advanced Cancer; Advanced squamous cell lung, pancreatic, head and neck, and other solid tumors	Intravenous PI3K/mTOR inhibitor	Phase 1 Phase 1	NCT01347866 NCT03065062
	PF-04691502	Breast Neoplasms	Inhibit PI3K and mTOR kinase	Phase 2	NCT01658176
	Vistusertib/AZD2014	Endometrial, triple negative breast cancer, ovarian, primary peritoneal, or fallopian tube cancer	mTORC1/2 Inhibitor	phase 1b/2	NCT02208375
AKT	MK-2206	Advanced or metastatic solid tumors or breast cancer; prostate cancer	Inhibit Akt phosphorylation, cell proliferation and apoptosis in a dose-dependent manner	Phase 1 Phase 2 Phase 2	NCT01245205 NCT01277757 NCT01251861
	Capivasertib/ AZD5363	Breast cancer, prostate cancer, and advanced solid tumors	A novel pan-AKT kinase catalytic inhibitor	Phase 1 Phase 1 Phase 2 Phase 1/2	NCT03310541 NCT01226316 NCT02525068 NCT01992952
	GSK2141795	Endometrial cancer	AKT inhibitor	Phase 1	NCT01935973
SREBP	Betulin	BRAF^V600E^-mutant melanoma	Increase membrane lipid poly-unsaturation and lipid peroxidation; sensitize therapy-resistant melanoma cells to MAPK-targeting therapy	Preclinical	(26)
	Fatostatin	Prostate cancer	A non-sterol diarylthiazole derivative which has antimitotic properties and perturbs nuclear translocation of SREBP and androgen receptor signaling	Preclinical	(109)
	PF-429242	HCC	A reversible site 1 protease inhibitors, which inhibits endogenous SREBP processing	Preclinical	(110)
AMPK	5-aminoimidazole 4-carboxamide riboside (AICAR)	Prostate cancer	An AMPK activator which inhibits cell growth	Preclinical	(111)
SCD1	A939572	Glioblastoma and renal cell carcinoma	Inhibit tumor growth both *in vitro* and *in vivo*; overcoming chemotherapy agent resistance	Preclinical	(112, 113)
	CVT-11127 or CVT-12012	Lung cancer	A small molecule SCD inhibitor which modulate cancer cell metabolism, proliferation and survival	Preclinical	(114)
	MF-438	Lung cancer	Induce lung cancer stem cells apoptosis	Preclinical	(115)
CPT1	Etomoxir	Glioma	A CPT1 inhibitor which inhibits proliferative activity	Preclinical	(41)
	Perhexiline	Breast and gastrointestinal cancer	A CPT1 inhibitor which blocks FFA utilization, OxPhos, and proliferation	Preclinical	(116, 117)
MAGL	Three different types of MAGL inhibitors	—	Potent and reversible MAGL inhibitors	Preclinical	(118–120)
HMGCR	Statin, *e.g.*, lovastatin, atorvastatin, rosuvastatin and simvastatin	Various leukemia and solid tumors	Inhibitors of HMGCR	Phase 2 Phase 2 Phase 2 Phase 2	NCT03358017 NCT03324425 NCT02569645 NCT03275376
LXR	GW3965; LXR623	Melanoma and glioblastoma	LXR agonists which suppress mitochondrial respiration and decrease cholesterol levels by enhancing the excretion and decreasing the resorption of cholesterol	Preclinical	(121)
ApoA-I	ApoA-I mimetic peptides	—	Mimetic peptides which is synthesized on the basis of α-amphipathic helical repeating structure of ApoA-I	Preclinical	(78)
	Apabetalone (RVX-208)	Colorectal cancer	A BET inhibitor which is a stimulator of ApoA-I and regulate the reverse cholesterol transport	Preclinical	(77)
ACAT	K604	Glioblastoma	A selective ACAT1 inhibitor, which suppresses proliferation of glioblastoma cells	Preclinical	(122)
	ATR-101	Advanced adrenocortical carcinoma	An oral selective ACAT inhibitor	Phase 1	NCT01898715 (123)

### Lipid Uptake Blocking

Orlistat, a compound that inhibits both LPL and FASN, may be used in tumors that are provided with LPL and express CD36 (8). However, the prolonged systemic suppression of LPL activity could result in hypertriglyceridemia and consequent pancreatitis, particularly if dietary fat intake is not curtailed. Furthermore, tumor cells may establish metastases in LPL-rich tissues, which can provide LPL to the nearby TME (8).

The potential of targeting oxLDL and related receptors (e.g., CD36 receptor) to reduce metastasis has been demonstrated in a variety of orthotopic cancer models (124). The direct impact of reducing oxLDL has never been investigated. At the same time, the use of anti-CD36 neutralizing antibodies causes almost complete inhibition of metastasis in immunodeficient or immunocompetent orthotopic mouse models of human oral cancer, with no side effects (125). CD36 monoclonal antibodies can reduce tumor growth in PCa patient-derived xenografts; at the same time, the combination with FASN inhibitor increases the efficacy of the CD36 blockade (99). In ovarian cancer, CD36 inhibition effectively reduced adhesion on collagen I matrices, which are abundantly expressed in the omental basement membrane where ovarian cancer cells preferentially attach (126). However, since CD36 also plays an important role in the myocardial metabolism of FAs (127), side effects caused by long-term inhibition of CD36 and the effect of direct targeting of oxLDL need further investigation.

### Targeting de Novo FA Biosynthesis

The inhibition of different enzymes (especially ACC and FASN, SCD) and the AKT/mTOR/SREBP-1 pathway within the *de novo* FA biosynthetic pathway can block cancer cell growth. Several ACC inhibitors, including TOFA, Soraphen A, and ND-646, have shown high efficacy in disturbing FAS, inducing oxidative stress, and inhibiting tumor growth in lung, colon, and prostate cancer in preclinical studies (104–106).

FASN is an established therapeutic target. First-generation (e.g., orlistat and cerulenin) and next-generation (TVB-3166 and TVB-2640) FASN-targeting drugs have been developed. Orlistat (tetrahydrolipstatin), a tight-binding irreversible inhibitor of the FASN thioesterase domain, exhibits both *in vitro* and *in vivo* antitumor properties against melanoma, breast cancer, PCa cells, and oral tongue squamous cell carcinoma (100). However, first-generation FASN inhibitors such as orlistat have pharmacological characteristics (e.g., lack of selectivity, poor metabolic stability, low cell permeability, etc.) and display detrimental systemic side effects (e.g., anorexia) (102). In contrast, next-generation FASN inhibitors exhibit anti-tumor potential, higher specificity for FASN, and limited systemic toxicity in a preclinical study. TVB-2640 was included in a phase II clinical trial. In multiple phases, 1/2 FASN inhibitor studies have been conducted (e.g., NCT00908791 for breast cancer, NCT02980029 for colon cancer, etc.), but no results have been published. TVB-3166 and TVB-3664 have shown anti-tumor activity in oral squamous cell carcinoma, breast, and colorectal cancer preclinical studies (102, 103). Giorgia et al. developed a selective and potent FASN inhibitor (IPI-9119), which can significantly reduce cell growth and induce cell cycle arrest and apoptosis in PCa cells, reduced growth of AR-V7-driven CRPC xenografts, and human mCRPC-derived organoids and enhances the efficacy of enzalutamide in CRPC cells (23). Furthermore, proton pump inhibitors (PPIs) are also effective inhibitors of the thioesterase activity of FASN (128), and a PPI, omeprazole, has entered clinical trials in patients with triple-negative breast cancer (NCT02595372).

The importance of the AKT/mTOR/SREBP-1 pathway in regulating gene expression of critical enzymes has received interest as a target. To date, several clinical trials aimed at evaluating different AKT inhibitors such as MK-2206 (NCT01245205, NCT01277757, NCT01251861), Capivasertib/AZD5363 (NCT03310541, NCT01226316, NCT02525068, NCT01992952), and GSK2141795 (NCT01935973) in multiple cancers; however, the results of these studies have not been reported to date. A combination of the mTOR inhibitor everolimus and metformin seems to synergistically inhibit proliferation and colony formation of breast cancer cells with few side effects *in vitro* (129). As an allosteric inhibitor of mTOR, some rapamycin analogs have been approved by the US Food and Drug Administration to treat breast cancer (107). Since knockdown and inhibition of SREBP-1 thwarts the glucose uptake, glycolytic activity, and lipid metabolism of HCC cells, betulin, a SREBP-1 inhibitor, has been shown to enhance the sensitivity of HCC cells to sorafenib (a first-line antitumor agent for advanced HCC treatment) and to facilitate its antitumor effect *in vivo*, suggesting that a SREBP-1 inhibitor and sorafenib combination can be a novel therapeutic option (25). In therapy-resistant melanoma, the inhibition of SREBP-1 re-sensitizes resistant cells to BRAF-targeting therapy *in vivo*, partly through alterations of membrane polyunsaturation and subsequent lipid peroxidation (26). Since SREBPs also induce androgen receptor (AR) activity in addition to lipogenesis, fatostatin, a non-sterol diarylthiazole derivative, has been reported to perturb the nuclear translocation of SREBP and caspase-dependent programed cell death of prostate cancer cells *in vitro* (109). Furthermore, AMPK inhibits SREBP activation through interaction with and phosphorylates SREBP-1 and SREBP-2 (111). Two agents that activate AMPK, 5-amino-4-imidazolecarboxamide ribose (AICAR) and rosiglitazone, reduce the activity of ACC and the concentrations of FASN and ACC in PCa cells and inhibit the stimulatory effect of androgen on these parameters (111).

Moreover, SCD1 appears to be a potential anticancer therapeutic target (37). A combined pharmacological approach involving SCD1 may counteract cancer cell chemoresistance and enhance the therapeutic efficacy of commonly used chemotherapeutic drugs, such as SCD1 inhibitor (CVT-11127 or CVT-12012) + gefitinib (114), SCD1 inhibitor (A939572) + temozolomide (112), SCD1 inhibitor (A939572) + temsirolimus (113). However, modest effects have been achieved by targeting SCD, suggesting that alternative desaturation pathways reduce the cancer cell dependence on SCD-mediated desaturation. Indeed, some cancer cell lines exploit an alternative FA desaturation pathway that desaturates palmitic acid to the unusual FA sapient *via* fatty acid desaturase 2 (FADS2) (130). Moreover, since SCD1 plays a major role in the *de novo* synthesis of TGs, cholesterol esters, and wax esters required for normal skin and eyelid function, the targeted disruption of *SCD1* in mice causes atrophy of sebaceous and meibomian glands and depletion of wax esters in the eyelid (131).

### Targeting FAO

FA catabolism inhibition might be a promising anticancer strategy. A blinded, placebo-controlled preclinical study showed that etomoxir, a CPT1 inhibitor, slowed tumor growth and prolonged survival in a mouse model of malignant glioma (41). Similarly, another β-oxidation inhibitor, perhexiline, blocked FFA utilization, OxPhos, and cancer cell proliferation *in vitro* (116). Recently, perhexiline was reported to increase the sensitivity of gastrointestinal cancer cells to oxaliplatin. This suggests that inhibiting FA catabolism can be a promising therapeutic strategy in combination with conventional chemotherapy for patients with gastrointestinal cancers (117).

### Targeting Lipolysis and Lipophagy

MAGL inhibition also decreases cyclin D1 and Bcl-2 expression, thereby inhibiting the proliferation and promoting tumor apoptosis or/and cell cycle arrest in endometrial cancer and CRC (132, 133). Three different types of MAGL inhibitors have been reported in the literature so far, which have been thoroughly reviewed by Carlotta et al. including (1): compounds that bind the enzyme covalently and irreversibly (2), compounds that bind it covalently and reversibly, and (3) compounds that bind it non-covalently (118). Irreversible covalent inhibitors are the most widespread class of MAGL inhibitors. In contrast, only recently, several reversible MAGL inhibitors have been reported to lack unwanted side effects from chronic treatment, including long-chain salicylketoxime derivatives and phenyl (piperazin-1-yl) methanone derivatives (118–120).

### Targeting Cholesterol Biosynthesis

Cancer cells need cholesterol for growth and survival and decreasing intracellular cholesterol biosynthesis may be a potential therapeutic strategy. Statins, inhibitors of HMGCR, trigger a robust homeostatic feedback response that ensures the cells upregulate and restore the MVA pathway, which has been successfully exploited for over 20 years to control hypercholesterolemia (82). Statins are proven to benefit lung cancer patients receiving EGFR-TKI therapy with improved response rates, prolonged progression-free survival, and overall survival by lowering cellular cholesterol, inducing loss of Cav1 expression, and triggering apoptosis to suppress NSCLC cell growth (91). Apart from cholesterol inhibition, statins inhibit cholesterol-independent processes, including cellular proliferation and intracellular signaling. However, statins were not associated with a reduced risk of pancreatic cancer in clinical trials. One possible reason is that HMGCR is also required for downstream protein prenylation, a critical process for protein activation. Thus, the statin effect not only inhibits cholesterol synthesis, but also other pathways that may render toxicity to normal cells. This nonspecific toxicity is a possible reason for the limited anticancer outcome of statins in clinical trials (94). At the same time, statins seem to increase the risk of some cancers, such as breast cancer and non-melanoma skin cancers, in several case-control studies and prospective studies (134). There are some serious adverse effects of statins such as muscle symptoms (e.g., rhabdomyolysis), type 2 diabetes mellitus, neurological and neurocognitive conditions (e.g., hemorrhagic stroke), hepatotoxicity, renal toxicity, and so on (135). Further investigation should focus on the more accurate and specific metabolite effects produced in the MVA pathway in different tumor cells.

### Targeting Cholesterol Transport

LXR agonists represent novel means to counteract cholesterol levels in tumor cells, including glioblastoma and melanoma, by enhancing the excretion (increase in ABCA1) and, at the same time, by decreasing the resorption of cholesterol (decrease in LDL receptor) (121). Since LXR agonists upregulate the expression of the pro-apoptotic Bcl-2 family member, Noxa, BH3 mimetics (ABT263 and ABT199), and LXR agonists synergistically reduce cellular viability by enhancing apoptosis, resulting in a synergistic anti-proliferative effect across solid tumor cells (121). In preclinical models of diffuse intrinsic pontine glioma, a small molecule, menin inhibitor MI-2, has been proven to disrupt cholesterol homeostasis by inhibiting the conversion of 2,3-oxidosqualene to lanosterol, which also activates LXR to increase cholesterol efflux (136).

Furthermore, some strategies aimed at directly augmenting ApoA-I (such as intravenous administration of autologous delipidated HDL, purified native ApoA-I, or recombinant ApoA-I Milano protein, etc.), or mimicking ApoA-I (such as ApoA-I mimetic peptides) functionality have already been used successfully in preclinical cancer studies (78).

### Targeting Cholesterol Esterification

In an orthotopic mouse model, cholesterol esterification inhibition disturbed cholesterol homeostasis by increasing the intracellular free cholesterol level, which was associated with elevated ER stress and eventually led to apoptosis and decreased tumor growth and metastasis (94). Several ACAT inhibitors, such as avasimibe, K604, and ATR-101, have been studied preclinically (123), and ATR-101 has entered clinical trials in patients with advanced adrenocortical carcinoma (NCT01898715).

## Conclusions and Prospective

Lipid metabolism not only composes the essential component of survival and proliferation in cancer cells, but also influences the crosstalk with immune cells in TME. With the gradual understanding of the mechanism of lipid metabolism in tumors, targeting the related enzymes and genes involved in the metabolism may provide an emerging approach for cancer treatment and restoration of tumor immunology.

However, not only tumor cells, but also immune cells, such as activated T cells, are considerable similarities in metabolic reprograming (137). The FA metabolic enzymes and synthetases are also involved in both cancer and normal whole-body metabolic homeostasis. For example, FASN expression is considered a metabolic marker of cell proliferation instead of merely a marker of malignancy (138). Therefore, by analyzing the difference of lipid content in tumor and normal tissue microenvironment, it is helpful to find more targeted lipid metabolism regulation strategies. At the same time, clarifying the specific mechanisms involved in the reprograming of lipid metabolism as well as their dual role in multiple tumor-associated signaling pathways may be better to identify therapeutic targets.

It is well known that some drugs have been used for metabolic diseases, such as FASN inhibitors, have been gradually developed for the field of anti-tumor therapy, and more overlap between metabolic diseases and cancer may be found in the future. So, additional investigations should carefully consider how to manage and combine targeting lipid metabolism or dietary interventions and other therapies to obtain the maximal clinical benefit.

## Author Contributions

WW and LB carried out the primary literature search, drafted and revised the manuscript, and participated in discussions. WL helped modify the manuscript. JC carried out the design of the research and literature analysis, drafted and revised the manuscript, and participated in discussions. All authors contributed to the article and approved the submitted version.

## Funding

This work was supported by the National Natural Science Foundation of China (Grant 81672275 and 81874052 to JC; grant 81702589 to LB); Project of Jilin Provincial Department of Education (Grant JJKH20190020KJ to JC); Project of Department of Science and Technology of Jilin Province (Grants 20180101009JC and 20190303146SF to JC); Outstanding Talent Cultivation Program for Doctoral Students in Norman Bethune Health Science Center of Jilin University (Grant to LB).

## Conflict of Interest

The authors declare that the research was conducted in the absence of any commercial or financial relationships that could be construed as a potential conflict of interest.

## Abbreviations

ATP, adenosine triphosphate; ACLY, ATP-citrate lyase; ACC, acetyl-CoA carboxylase; ACACA, CoA carboxylase; ATGL, adipose triglyceride lipase; ApoA-I, apolipoprotein A-I; ABCA1, ATP-binding cassette transporter; ACAT, acyl-CoA cholesterol acyltransferase; AR, androgen receptor; CSCs, cancer stem-like cells; CEs, cholesteryl esters; CPT, carnitine palmitoyltransferase; CAT, carnitine-acylcarnitine translocase; ccRCC, clear cell renal cell carcinoma; CRC, colorectal cancer; CYP27A1, Cytochrome P450 Family 27 Subfamily A Member 1; EMT, epithelial-mesenchymal transition; EGFR, epidermal growth factor receptor; ERK, extracellular signal-regulated kinases; ER, estrogen receptor; FAs, fatty acids; FAS, fatty acid synthesis; FASN, fatty acid synthase; FAO, fatty acid oxidation; FATPs, fatty acid transport protein family; FABPpm, plasma membrane fatty acid-binding proteins; HCC, hepatocellular carcinoma; HIF, hypoxia inducible factor; HSL, hormone-sensitive lipase; HDL, high density lipoprotein; HMGCR, 3-Hydroxy-3-methylglutarylcoenzyme A reductase; HMG-CoA, 3-Hydroxy-3-methylglutarylcoenzyme A; HSF1, heat shock factor 1; IGF, insulinlike growth factor; LPCAT1, lysophosphatidylcholine-acyltransferase 1; LPL, lipoprotein lipase; LDs, lipid droplets; LDL, low density lipoprotein; LXR, liver-X-receptor; MUFA, mono-unsaturated fatty acids; mCRPC, metastatic castration-resistant prostate cancer; MAGL, monoacylglycerol lipase; NADPH, nicotinamide adenine dinucleotide phosphate; NSCLC, non-small cell lung cancer; OXPHOS, oxidative phosphorylation; PLs, phospholipids; PI3K, phosphoinositide 3-kinase; PCa, prostate cancer; ROS, reactive oxygen species; SCD, stearoyl-CoA desaturase; SREBP, sterol regulatory element binding proteins; SFAs, saturated fatty acids; SCAP, SREBP cleavage activated protein; SR-B1, scavenger receptor class B type 1; TME, tumor microenvironment; TG, triglyceride; VLDL, very-low-density lipoprotein; 27-HC, 27-hydroxycholesterol.
